# Cognitive Behavioural Therapy through PowerPoint: Efficacy in an Adolescent Clinical Population with Depression and Anxiety

**DOI:** 10.1155/2018/1396216

**Published:** 2018-11-08

**Authors:** Nazanin Alavi, Matthew Stefanoff, Alyssa Hirji, Sarosh Khalid-Khan

**Affiliations:** ^1^Department of Psychiatry, Queen's University, Providence Care Hospital, 752 King Street West, Postal Bag 603, Kingston, Ontario, K7L 7X3, Canada; ^2^Department of Psychiatry, University of Toronto, Centre for Addiction and Mental Health, 100 Stokes Street, Toronto, Ontario, M6J 1H4, Canada; ^3^Department of Psychology, Queen's University, Humphrey Hall, Room 232, Queen's University, Kingston, Ontario, K7L 2N6, Canada

## Abstract

**Background:**

Limited help-seeking behaviours, among adolescents with mental health concerns and many barriers to accessing mental health services, make innovative approaches to administering mental health therapies crucial. Therefore, this study evaluated the efficacy of e-CBT given via PowerPoint slides to treat adolescents with anxiety and/or depression.

**Method:**

15 adolescents referred to an outpatient adolescent psychiatry clinic to treat a primary DSM-IV diagnosis of anxiety and/or depression chose between 8 weeks of e-CBT (n=7) or 7 weeks of live CBT (n=8). The e-CBT modules were presented using PowerPoint delivered weekly through email by either a senior psychiatry resident or an attending physician. Within each session, participants in both groups had personalized feedback on their mandatory weekly homework assignment from the previous week's module. BYIs were completed before treatment and and after final treatment within both groups to assess changes in depression, anxiety, anger, disruption, and self-concept.

**Findings:**

Before treatment, BYI scores did not sig. differ between groups. After treatment, e-CBT participants reported sig. improved depression, anger, anxiety, and self-concept BYI scores while live CBT participants did not report any sig. changes. Only the Beck Anxiety Inventory sig. differed between groups after CBT.

**Conclusion:**

Despite the low sample size within this study, using email to deliver e-CBT PowerPoint slides and individualized homework feedback shows promise as an alternate method of CBT delivery that reduces barriers to receiving mental health treatment that occur internationally.

## 1. Introduction

Mental illness is the leading cause of disability among adolescents worldwide [1]. In an American study, it was found that 50% of adolescents met the diagnostic criteria for at least one DSM-IV disorder and 28% of the sample experienced a severe impairment [2]. About 1/3 of the adolescents met the diagnostic criteria for an anxiety disorder and 14% met the criteria for a mood disorder [2]. If left untreated, anxiety and depression can place a significant burden on the economy, impair individuals' daily functioning, and reduce quality of life [3, 4]. CBT has effectively improved both anxiety and depression and so minimizes the strain of these disorders on economic and personal levels. As every 1 in 4-5 adolescents has a chronic psychiatric disorder [2], CBT may be particularly important for this young population, as early therapy is associated with a better long-term prognosis [5].

Despite the efficacy of CBT and the benefits of early intervention, only 44% and 15% of adolescents with depressive symptoms receive treatment in developed and developing countries, respectively [2, 6]. Living in a rural area, a lack of mental health resources and/or accessibility, and doubt over the confidentiality of treatment perpetuated by negative stigmas have all been said to negatively impact the availability and pursuit of treatment [7, 8]. To combat these barriers, there has been a surge in the examination of innovative treatments, many involving technology and the internet.

With over 2.5 billion people worldwide using the internet [9], the research, development, and use of computer-based CBT (e-CBT) has been booming due to its potential to reduce many barriers of traditional face-to-face therapies [10]. E-CBT is very cost-effective and increases treatment accessibility and adherence to individuals where mental health resources are lacking [11, 12]. Many patients have reported e-CBT as preferable due to its convenience, confidentiality, and reduction of perceived stigma [10]. Finally, e-CBT has many practical advantages, such as the individualization of programs and therefore treatments, along with self-pacing, the ability to review material, and the ease of record keeping and data collection [10]. As most adolescents are familiar and comfortable with computers and the internet, e-CBT may be particularly effective for treating mental health concerns of this age.

While various programs for the online treatment of depression and anxiety exist, many do not have an efficacy comparable to live treatment. For example, MoodGYM is a current e-CBT based on cognitive restructuring, pleasant events scheduling, and interpersonal problem solving [8]. It allows the participant to work through material at their own pace and consists of 5 sessions to be completed over 3 weeks. In a study of the efficacy of MoodGYM, adolescent 1st year undergraduates with anxiety or depression were randomised to either live CBT, MoodGYM, both combined, or no treatment groups. All treatment groups had a reduction in both depression and anxiety compared to the control group, but the live CBT group had a greater decline in depression than those using MoodGYM. Also, the combined group had significantly lower anxiety and depression after the intervention than the MoodGYM only group [8]. The authors suggest that these findings may be due to MoodGYM being unsuitable for this age group because of the complexity of the therapy components or the slow pace of the program [8].

Another program, BRAVE for Teenagers-Online, used for adolescents with anxiety disorders consists of 10 one-hour long sessions which replicate the clinic-based version of the program while incorporating standard CBT anxiety management strategies [13]. Participants were assigned homework at the end of each session and had a BRAVE trainer who monitored their work, gave support, and feedback. All participants were randomised to live CBT, e-CBT, or a wait-list control. Significantly more participants in the treatment groups no longer met the criteria for an anxiety diagnosis after treatment than in the control group, with no differences between the live and e-CBT groups on efficacy or participant satisfaction [13]. The % of adolescents with an anxiety disorder continued to fall in both groups at the 6 and 12 month follow-ups, with no sig. differences between the treatment groups [13]. Taken together, the MoodGYM and BRAVE findings suggest that e-CBT may be as effective as live CBT, but not all e-CBT programs are equivalent.

This study examines the efficacy of e-CBT delivered through PowerPoint slides combined with weekly computer-based psychiatrist-given feedback to adolescents with anxiety or depression; this study is the first to examine efficacy using this treatment modality. We hypothesized that e-CBT would be as efficacious as live CBT in improving depression and anxiety.

## 2. Materials & Methods

### 2.1. Participants

Adolescent males (*n* = 2) and females (*n* = 13) aged 14-17 (*M*=14.7,* SD*=1.50) who met DSM-IV criteria for major depressive disorder and generalized anxiety disorder were invited to participate in the study. Participants were recruited from the outpatient Child and Youth Mental Health Program at the Hotel Dieu Hospital site of the Kingston Health Sciences Centre in Kingston, Ontario. Each participant was recommended to participate in CBT for the treatment of their primary diagnosis.

### 2.2. Measures

Participants were assessed using the Beck Youth Inventories (BYI), a self-report instrument used to assess youth aged 7-18. The measure consists of 5 composite scores: the Beck Depression Inventory for Youth (BDI-Y), the Beck Anxiety Inventory for Youth (BAI-Y), the Beck Anger Inventory for Youth (BANI-Y), the Beck Disruptive Inventory for Youth (BDBI-Y), and the Beck Self-Concept Inventory for Youth (BSCI-Y). Each inventory consists of 20 items rated on a 4-point scale from 0 (never) to 3 (always). Total raw scores can range from 0 to 60, with higher scores representing more of the construct.

### 2.3. Procedure

To maintain a naturalistic study design, each participant chose between live and e-CBT. The e-CBT sessions were designed to directly mirror the live CBT sessions, but live CBT consisted of 7 sessions, whereas the e-CBT consisted of 8. The 8th e-CBT session only collected feedback without giving any new content, so the live and e-CBT sessions were considered to be equivalent.

For each online session, participants were sent about 20-30 PowerPoint slides every Wednesday, consisting of general information on a weekly topic, an overview of helpful skills that relate to the weekly topic, and mandatory homework sheets (Table 1). The homework assignments were due every Sunday. They were received by either a senior psychiatry resident or attending physician, who sent each e-CBT participant individualized feedback, the next session's slides, and corresponding homework via email on the next Wednesday. Homework completion and submission was mandatory to progress to the next week's session. If homework was not returned by the deadline, a reminder email was sent. The control group had weekly one-hour live CBT sessions, with matching homework assignments.

The BYI was completed by all participants both before treatment and after completing their final CBT session. All BYIs were completed within appointments in the clinic for both live CBT and e-CBT groups.

Descriptive statistics and independent sample t-tests were used to examine demographic differences between groups, while repeated measures analysis of variance (ANOVA) testing was used to determine the effect of time and treatment modality on each BYI inventory. Pairwise comparisons were conducted using a Bonferroni adjustment and all analyses were done using SPSS.

This study was reviewed for ethical compliance by the Research and Ethics Board of Queen's University, Canada.

## 3. Results

Of the 15 study participants, 7 chose e-CBT and 8 chose live CBT. Age was not significantly different between groups (*t*(13)=-2.044,* p*=0.062) with the mean age of e-CBT and live CBT groups being 15.4 (*SD*=1.27) and 14.0 (*SD*=0.50), respectively. Each group had 1 male participant, 6 females had e-CBT, and 7 had live CBT. Before treatment, the 2 groups did not differ significantly on any BYI scores (Table 2).

### 3.1. BDI-Y

The 2 groups did not differ significantly (*F*(1,13)=0.40,* p*=0.54) in BDI-Y scores after treatment: the e-CBT group had a mean score of 18.7 (*SD*=14.91) and the live CBT group a mean score of 23.3 (*SD*=18.71). Pre- and posttreatment BDI-Y scores did not differ significantly within the live CBT group (*F*(1,13)=0.87,* p*=0.37), but within the e-CBT group they fell significantly over time (*F*(1,13)=7.82,* p*=0.02).

### 3.2. BAI-Y

BAI-Y scores after treatment were significantly lower (*F*(1, 13)=7.04,* p*=0.02) in the e-CBT group (*M*=13.1,* SD*=10.49) than the live CBT group (*M*=27.3,* SD*=10.08). Additionally, pre- and posttreatment BAI-Y scores within the live CBT group did not change significantly (*F*(1,13)=0.22,* p*=0.64), whereas the e-CBT group BAI-Y scores fell significantly over time (*F*(1,13)=10.55,* p*=0.01).

### 3.3. BANI-Y

The e-CBT (*M*=17.1,* SD*=16.23) and live CBT (*M*=27.5,* SD*=12.58) groups did not significantly differ (*F*(1,13)=1.94,* p*=0.19) on posttreatment BANI-Y scores. The pre- and posttreatment BANI-Y scores did not differ significantly within the live CBT group (*F*(1,13)=1.61,* p*=0.23), but BANI-Y scores within the e-CBT group fell significantly over time (*F*(1,13)=5.20,* p*=0.04).

### 3.4. BDBI-Y

The 2 groups did not differ significantly (*F*(1,13) =0.83* p*=0.38) in BDBI-Y scores after treatment, with e-CBT having a mean score of 5.7 (SD=7.9) and live CBT a mean of 9.5 (*SD*=8.11). The pre- and post-treatment BDBI-Y scores did not differ significantly over time within the live CBT group (*F*(1,13)=4.53,* p*=0.53) or within the e-CBT group (*F*(1,13)=4.03,* p*=0.07).

### 3.5. BSCI-Y

The e-CBT (*M*=29.7,* SD*=12.67) and live CBT (*M*=28.5,* SD*=11.83) groups did not differ significantly (*F*(1, 13) =0.04,* p*=0.85) on posttreatment BSCI-Y scores. The pre- and posttreatment BSCI-Y scores did not differ significantly within the live CBT group (*F*(1,13)=0.57,* p*=0.46), but the e-CBT BSCI-Y scores rose significantly over time (*F*(1,13)=5.57,* p*=0.04).

## 4. Discussion

Results of this study suggest that e-CBT delivered via PowerPoint is effective for improving depression and anxiety in adolescents. Contrary to expectations, e-CBT via PowerPoint with clinician-provided feedback may be more effective than live CBT in reducing symptoms. The e-CBT group improved significantly in anxiety, depression, anger, and self-concept after treatment, whereas the live CBT group did not improve significantly over time in any of the BYI inventories. Despite the significant changes in the e-CBT group within 4 of the 5 BYI inventories, the two groups only differed significantly posttreatment in anxiety, with e-CBT having significantly more reduction in BAI scores than live CBT. The significance of e-CBT on BAI scores is important as anxiety disorders affect 1/3 of adolescents [2], and unmanaged anxiety disorders in adolescents correlate with an increased risk of illicit drug dependence, depression, and academic underachievement [14].

The study's findings are unique in that the e-CBT sessions related to significant improvements in two inventories not directly related to the adolescents' primary diagnoses: anger and self-concept. This suggests that the topics covered within the e-CBT modules improve not only self-reported depression and anxiety but also other problems with which youth presenting to an outpatient psychiatric clinic may have difficulties. Surprisingly, however, this effect was only seen when the modules were given via PowerPoint e-CBT.

These findings are inconsistent with previous findings of live CBT being more effective than e-CBT for depression and/or anxiety in adolescents [8, 11]. This may be due to the use of standardized e-CBT programs in previous studies, whereas the current study used weekly PowerPoint slides with corresponding individualized psychiatrist-provided feedback. This personalized feedback allows for increased communication between therapist and client via email and so more closely resembles traditional face-to-face therapy. This personalization of treatment may help explain why this intervention was more effective than other online CBT programs.

While older research emphasized the negative aspects of adolescents using the internet, more recently, it was found to be beneficial [15]. Since many adolescents are wary of seeking treatment for mental health concerns, the existence of a more accessible virtual treatment modality may increase adolescents' willingness to both seek out and participate in treatment [16]. Within this study, a decreased reluctance to participate in therapy could have led to an increased engagement with treatment in the e-CBT group, further contributing to the increased efficacy of e-CBT compared to live CBT.

This study was limited by the small sample size and lack of treatment modality randomization. The absence of randomization may introduce additional confounding variables. However, by giving patients the option to choose their CBT delivery method, this study is a naturalistic approach to examining efficacy by mirroring the decisions made in clinical settings. Also, participants' comfort with using computers, emails, and PowerPoint may have influenced the efficacy of e-CBT [17]. Since no measures were employed to evaluate comfort with these technologies and programs, this is a possible confounder and so it is likely that those comfortable with computers chose the e-CBT option, potentially inflating the results of that group.

Future research should determine the effects of familiarity with computers as a confounding variable on the efficacy of e-CBT, in addition to randomizing the treatment modality between patients to determine if the results of this study were influenced by patients' ability to choose their method of receiving CBT. Further studies of the efficacy of e-CBT via PowerPoint in adolescents would also benefit from a longer follow-up period to determine the duration of the treatment effects. However, previous studies examining e-CBT via PowerPoint for depression given to adults were immediately effective and the results were evident at the 6-month follow-up [12].

## 5. Conclusion

While further studies are warranted due to the small sample size of the current study, they are the first to show that computer-based CBT via PowerPoint slides may be an effective way to improve depression and anxiety in adolescents. This simple, innovative and user-friendly way to deliver CBT to adolescents might reduce barriers to treatment such as lack of resources, missing class for appointments, and the high costs of software development. It may be particularly beneficial for adolescents comfortable with technology who may be concerned with stigma associated with attending live CBT, by allowing treatment to be completed at home. Also, this method of e-CBT may be more effective than other standardized e-CBT programs previously examined because it can be quickly and easily tailored to meet the needs of each individual patient. Not only can the therapist address individual concerns or elaborate on material when needed, but e-CBT also can be easily adapted for other languages or cultures that have specific needs. Thus, e-CBT via PowerPoint is an innovative therapy that has promise as a new way to deliver CBT to improve adolescent depression and anxiety and can remove barriers that prevent youth from receiving mental health treatment.

## Figures and Tables

**Table 1:** CBT topics by week of administration.

Week	Topics Covered
1	Introduction, Goals
2	Five Party Model
3	Thoughts
4	Connection between Thoughts, feelings, Behaviour, Physical Reactions and Environment
5	‘Evidence' and ‘Alternative and Balanced Thinking'
6	Experiments and Action Plans
7	Strategies to overcome the distress
8^*∗*^	Summary and Feedback

^*∗*^ Module only given to e-CBT participants.

**Table 2:** Group difference in BYI scales before receiving any CBT.

	e-CBT (n=7)		Live (n=8)				
BYI Scale	M	SD	M	SD	*t*	*df*	*p*
BDI-Y	24.7	16.98	21.4	12.72	0.44	13	0.67
BAI-Y	19.6	12.34	28.1	13.39	-1.28	13	0.22
BANI-Y	22.4	17.53	24.8	13.58	-0.29	13	0.77
BDBI-Y	7.9	9.97	7.4	7.48	0.11	13	0.92
BSCI-Y	24.3	12.71	30.1	9,73	-1.01	13	0.33

## Data Availability

The data used to support the findings of this study are available in excel format in the following link: https://docs.google.com/spreadsheets/d/1yLsvPOLXcERXd_rA6uoTNbnkwohAh2AQxYzI639FPKA/edit?usp=sharing. The data can also be obtained by the corresponding author upon request.

## References

[1] Erskine H. E., Moffitt T. E., Copeland W. E. (2015). A heavy burden on young minds: The global burden of mental and substance use disorders in children and youth. *Psychological Medicine*.

[2] Merikangas K. R., He J.-P., Burstein M. (2010). Lifetime prevalence of mental disorders in U.S. adolescents: results from the national comorbidity survey replication-adolescent supplement (NCS-A). *Journal of the American Academy of Child & Adolescent Psychiatry*.

[3] Hoffman D. L., Dukes E. M., Wittchen H.-U. (2008). Human and economic burden of generalized anxiety disorder. *Depression and Anxiety*.

[4] Ruwaard J., Schrieken B., Schrijver M. (2009). Standardized web-based cognitive behavioural therapy of mild to moderate depression: A randomized controlled trial with a long-term follow-up. *Cognitive Behaviour Therapy*.

[5] Guralnick M. J. (1998). Effectiveness of early intervention for vulnerable children: A developmental perspective. *American journal of Mental Retardation*.

[6] Arnberg F. K., Linton S. J., Hultcrantz M., Heintz E., Jonsson U. (2014). Internet-delivered psychological treatments for mood and anxiety disorders: A systematic review of their efficacy, safety, and cost-effectiveness. *PLoS ONE*.

[7] Shafran R., Clark D. M., Fairburn C. G. (2009). Mind the gap: improving the dissemination of CBT. *Behaviour Research and Therapy*.

[8] Sethi S., Campbell A. J., Ellis L. A. (2010). The use of computerized self-help packages to treat adolescent depression and anxiety. *Journal of Technology in Human Services*.

[9] Chen C.-C., Wang C.-C. (2013). Internet as indispensable everywhere: The introduction to the advances in internet technologies and applications special issue. *Journal of Computers (Finland)*.

[10] Marks I., Cavanagh K. (2009). Computer-aided psychological treatments: Evolving issues. *Annual Review of Clinical Psychology*.

[11] Khanna M. S., Kendall P. C. (2010). Computer-assisted cognitive behavioral therapy for child anxiety: Results of a randomized clinical trial. *Journal of Consulting and Clinical Psychology*.

[12] Alavi N., Hirji A., Sutton C., Naeem F. (2016). Online CBT Is Effective in Overcoming Cultural and Language Barriers in Patients with Depression. *Journal of Psychiatric Practice*.

[13] Spence S. H., Donovan C. L., March S. (2011). A randomized controlled trial of online versus clinic-based CBT for adolescent anxiety. *Journal of Consulting and Clinical Psychology*.

[14] Woodward L. J., Fergusson D. M. (2001). Life course outcomes of young people with anxiety disorders in adolescence. *Journal of the American Academy of Child and Adolescent Psychiatry*.

[15] Valkenburg P. M., Peter J. (2009). Social consequences of the Internet for adolescents: A decade of research. *Current Directions in Psychological Science*.

[16] Zachrisson H. D., Rödje K., Mykletun A. (2006). Utilization of health services in relation to mental health problems in adolescents: A population based survey. *BMC Public Health*.

[17] Mitchell N., Gordon P. K. (2007). Attitudes towards computerized cbt for depression amongst a student population. *Behavioural and Cognitive Psychotherapy*.

